# Combined effects of agitation and aeration on the chitinolytic enzymes production by the Antarctic fungus *Lecanicillium muscarium *CCFEE 5003

**DOI:** 10.1186/1475-2859-11-12

**Published:** 2012-01-23

**Authors:** Massimiliano Fenice, Paolo Barghini, Laura Selbmann, Federico Federici

**Affiliations:** 1 Dipartimento di Scienze Ecologiche e Biologiche, Largo Università snc, University of Tuscia, I-01100, Viterbo, Italy

**Keywords:** Chitinolytic enzymes production, *Lecanicillium muscarium*, Response Surface Methodology, Agitation and aeration

## Abstract

**Background:**

The Antarctic fungus *Lecanicillium muscarium *CCFEE 5003 is one of the most powerful chitinolytic organisms. It can produce high level of chitinolytic enzymes in a wide range of temperatures (5-30°C). Chitinolytic enzymes have lot of applications but their industrial production is still rather limited and no cold-active enzymes are produced. In view of massive production of *L. muscarium *chitinolytic enzymes, its cultivation in bioreactors is mandatory. Microbial cultivation and/or their metabolite production in bioreactors are sometime not possible and must be verified and optimized for possible exploitation. Agitation and aeration are the most important parameters in order to allow process up-scaling to the industrial level.

**Results:**

In this study, submerged cultures of *L. muscarium *CCFEE 5003 were carried out in a 2-L bench-top CSTR bioreactor in order to optimise the production of chitinolytic enzymes. The effect of stirrer speed (range 200-500 rpm) and aeration rate (range 0.5-1.5 vvm) combination was studied, by Response Surface Methodology (RSM), in a medium containing 1.0% yeast nitrogen base and 1% colloidal chitin. Optimization was carried out, within a "quadratic D-optimal" model, using quantitative and quantitative-multilevel factors for aeration and agitation, respectively. The model showed very good correlation parameters (R^2^, 0.931; Q^2^, 0.869) and the maximum of activity (373.0 U/L) was predicted at ca. 327 rpm and 1.1 vvm. However, the experimental data showed that highest activity (383.7 ± 7.8 U/L) was recorded at 1 vvm and 300 rpm. Evident shear effect caused by stirrer speed and, partially, by high aeration rates were observed. Under optimized conditions in bioreactor the fungus was able to produce a higher number of chitinolytic enzymes than those released in shaken flasks. In addition, production was 23% higher.

**Conclusions:**

This work demonstrated the attitude of *L. muscarium *CCFEE 5003 to grow in bench-top bioreactor; outlined the strong influence of aeration and agitation on its growth and enzyme production and identified the optimal conditions for possible production at the industrial level.

## Background

Chitinolytic enzymes (generally called chitinases) have been widely studied and some produced by fungi are of great interest for possible applications. Traditionally, these hydrolases could be employed in chitin hydrolysis, production of chitin derivatives, protoplast formation and bio-control of pathogenic organisms [1-4]. Some unconventional and very interesting applications in food and wine industries have been successfully tested at laboratory level [5,6]. In this context, the search of new chitinolytic organisms and/or enzymes is still full of interest. In addition, in some specific fields, chitinolytic cold tolerant microorganisms or enzymes could solve a number of practical problems.

The production of chitinases at industrial level is rather scarce and mainly obtained from *Serratia marcescens *and *Streptomyces griseus; *the only commercial chitinase preparation of fungal origin comes from selected strains of *Trichoderma harzianum*. However, the cost of these enzymes is still too high and large scale applications are still expensive and scarcely profitable. Already from their early works, Aloise et al. [7] proposed an industrial process to obtain *N*-acetyl-D-glucosamine (NAG) using *S. marcescens *chitinolytic enzyme.

So far, however, no process, using fungi and/or their enzymes, has been studied in details in view of industrial scale-up.

In this context, setting of best bioprocess (fermentation) conditions is very important and, in particular, understanding the effect of agitation/aeration on microbial productions is generally recognized as a mandatory key step for the scale-up to the industrial level.

The Antarctic fungus *Lecanicillium muscarium *CCFEE 5003, belonging to a species generally recognized as entomopathogenic, produces high levels of chitinases, when grown on raw or colloidal chitin, in a wide range (5-30°C) of temperatures with an optimum at 25°C [8,9]. The chitinolytic activity of this psychrotolerant organism, also related to its ability to attack and destroy other fungal organisms (mycoparasitism), is associated to the production of various enzymes including chitinases, glucanases and proteases [3,9]. Strain CCFEE 5003 is really very powerful at low temperature. It is even more efficient than *T. harzianum *that is commercialized as a bio-control mycoparasitic agent against plant pathogens but shows scarce activity at low temperature and in cold environments [10,11].

In view of potential applications of both the fungus and its chitinases, massive (industrial) optimized cultivation is necessary: this could only occur in bioreactors. However, since sometimes microorganism's growth in bioreactors is scarce and their release of metabolites fails, the capacity to growth and produce in such conditions must be proved [12].

It is worth noting that, nowadays, it is generally recognized that optimization of microbial production process should be performed using fast, efficient and correctly designed model-based methods [13]. Unfortunately, many recent publications show that scientists continue to employ techniques that permit to vary "one-variable-at-a-time" (OVT), only. This does not consent to elucidate the combined effect of the various parameters and underline which factors could have significant interactions (synergy or antagonism). Moreover, it is not possible to understand which variables have real influence on the response (result) and to predict the effect exerted by the combined variation of the factors. This happens also for the optimization of fungal chitinases production. In fact, till now modeling has been used for process optimization in shaken flasks only, while in bioreactor optimization has been carried by the OVT technique [14-18]. In addition, most of the works regarded process optimization for *Trichoderma *species; the only paper available for the optimization of chitinolytic enzymes by *L. muscarium *is that of Liu et al. [15] that was carried out with traditional methods (OVT).

In this paper we studied by RSM the combined effects of agitation and aeration on the production of chitinolytic enzymes by *L. muscarium *CCFEE 5003, cultivated in a 2-l bench-top CSTR bioreactor, in view of possible scale-up to the industrial level. In this context, effects of these parameters on the volumetric mass-transfer coefficient (K_L_a) and time course of chitinolytic enzymes production and main bioprocess variables under optimized conditions are also reported.

## Results and discussion

Experimental design is the investigation planning carried out in order to extract the maximum amount of information form the collected data by performing the minimum number of experiments. The idea is to vary all relevant factors simultaneously over a set of defined experiments and to connect the results by a mathematical model that is used for data interpretation, prediction and optimization. The evolution of statistical experimental designs, started with the very early works of Fisher in the twenties of last century and refined by others, provides the user with powerful methodologies for efficient experimentation [19-21].

RSM, one of the most efficient and performing methods for experimental design, is largely used in fermentation technology to optimize process parameters both at laboratory and industrial level [22,23]. However, for the optimization of fungal chitinases production, this method has been used only for experiments in shaken flasks [14,18]. As far as we know, this is the first paper dealing with the optimization of agitation and aeration in CSTR for the production of fungal chitinases by RSM. Moreover, this is the first work assessing the ability of *L. muscarium *to grow and produce high level of chitinolytic enzymes in stirred bioreactors.

### Model performance

Data were best fitted by a polynomial quadratic equation, as it can be inferred by the good agreement of experimental data with those estimated by the model.

As indicated by the Anova table, the model revealed high reliability and good statistical performance (Table 1). For both the analysed responses, the probability for the regression was significant at 95% and there was no lack of fit.

**Table 1:** Statistical parameter measuring the correlation and significance of the model (Anova table)

Enzyme activity	DF	SS	MS	F	p	SD
Total	20	1.12 × 10^6^	56003			
Constant	1	920467	920467			
Total (Corrected)	19	199593	10504.9			102.493
Regression	5	185766	37153.2	37.6185	0	192.752
Residual	14	13826.8	987.63			31.4266
Lack of Fit (model error)	6	8443.66	1407.28	2.09138	0.165	37.5137
Pure Error (replicate error)	8	5383.16	672.895			25.9402
**N **= 20	Q^2 ^= 0.869		Cond. no. = 3.922			
DF = 14	R^2 ^= 0.931		Y-miss = 0			
	R^2 ^Adj. = 0.906		RSD = 31.4266			
						
K_L_a						
Total	20	55583.6	2779.18			
Constant	1	49658.6	49658.6			
Total (Corrected)	19	5925.00	311.842			17.659
Regression	5	5754.58	1150.92	94.5509	0	33.9252
Residual	14	170.414	12.1724			3.4889
Lack of Fit (model error)	6	11.981	18.4969	2.48978	0.116	4.3008
Pure Error (replicate error)	8	59.4329	7.42911			2.72564
**N **= 20	Q^2 ^= 0.947		Cond. no. = 3.922			
DF = 14	R^2 ^= 0.971		Y-miss = 0			
	R^2 ^Adj. = 0.961		RSD = 3.4889			

Legend DF, degree of freedom; SS, sum of squares; MS, Mean Square (SS/DS); F, F-distribution value; *p*, probability; SD, standard deviation

As for the enzyme activity, the correlation coefficient (R^2^), indicating the fraction of response variation explained by the model, was high (0.931). This means that the statistical model can explain 93.1% of response variability. Also Q^2^, indicating the fraction of response variation that can be predicted by the model, was rather good (0.870). Finally, the rather high F-value (37.6) indicated that the model terms were quite significant.

For K_L_a, statistical parameters were even better being R^2 ^0.971, Q^2 ^0.947 and F-value 99.6, respectively.

### Effects of aeration and agitation on K_L_a and enzyme production

Oxygen must be supplied to all aerobic cultures to satisfy the request for growth and production. In CSTRs this is mainly obtained by correct set up of aeration and agitation leading to the transfer of a sufficient oxygen amount to each cell. K_L_a is the most significant parameter to measure transfer phenomena, including oxygen transfer, inside a bioreactor. K_L_a could be improved by increasing aeration and/or agitation but with technical and physiological (shear stress) limitations.

**Figure 1:**
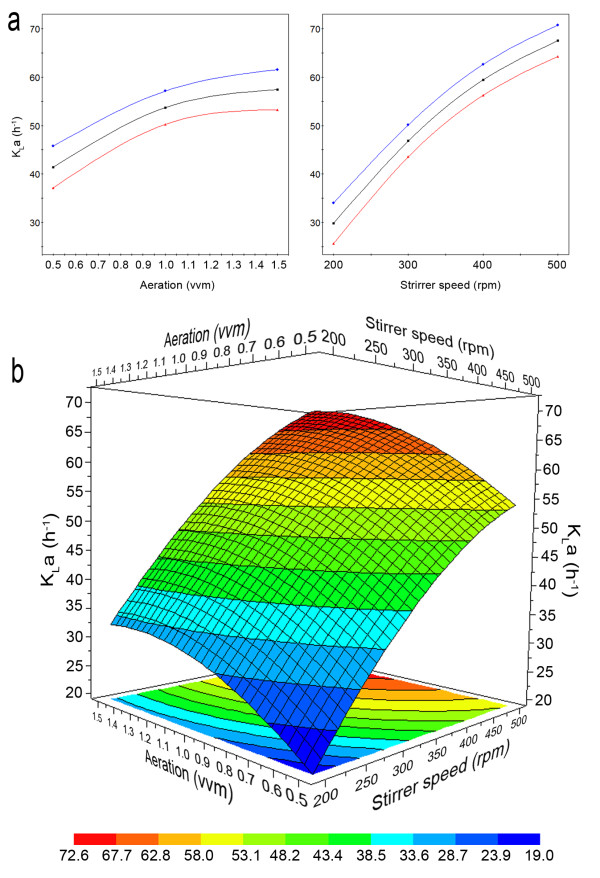
**Single (a) and combined (b) effect of agitation and aeration on K_L_a measured in 2 L CSTR as predicted by RSM**. (a) Prediction plot: average, minimum and maximum predicted values (confidence level, 95%) were represented by the black, red and blue lines, respectively. (b) Surface response: color codes indicated different ranges of values.

Figure 1 shows K_L_a values measured at various combinations of stirrer speed and aeration rate as suggested by the model.

The increase of both parameters, in all cases, led to increased K_L_a. However, considering the single parameter effect, agitation was much more effective than aeration, being its relative curve much more steep (Figure 1a).

This was even more evident considering the combined effect plotted in the response surface (Figure 1b). At 200 rpm, K_L_a increase was limited for every aeration rates. The stirrer was barely able to load the air flow ("loading") with consequent little air dispersion. Evident "flooding" (unperturbed air passage through the stirrer) was recorded in particular at highest flow rates. K_L_a measured at lowest aeration and highest agitation was much higher (ca. 54 h^-1^) than that in opposite conditions (ca. 30 h^-1^). It is worth noting that K_L_a increasing rate (curve slope) at the highest values of both parameters was lower than that recorded at the lowest values. In fact, the response surface in the lower part of the plot (blue - dark green coded colours) was quite steep, while in the intermediate part (light green - orange colours) it started to flatten reaching almost a plateau thereafter (red zone). This could be due to possible saturation effect.

**Figure 2:**
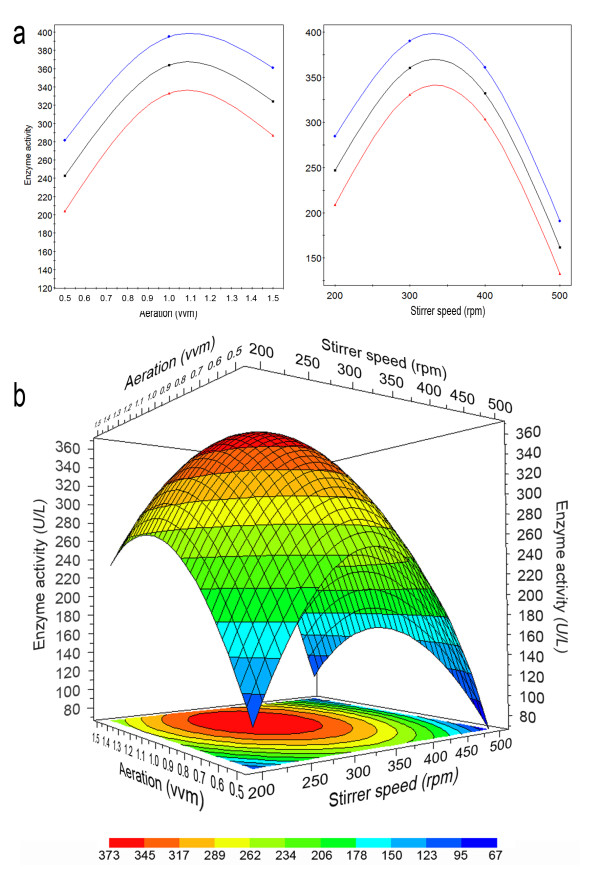
**Single (a) and combined (b) effect of agitation and aeration on the total chitinolytic activity of *L. muscarium *CCFEE 5003 cultivated in 2 L CSTR as predicted by RSM**. (a) Prediction plot: average, minimum and maximum predicted values (confidence level, 95%) were represented by the black, red and blue lines, respectively. (b) Surface response: color codes indicated different ranges of values.

As expected, both aeration and agitation heavily influenced *L. muscarium *chitinolytic activity. However, unlike that occurred for K_L_a, positive effects were observed till a threshold level only, after which negative effects started. This was evident either when the factors where considered alone or in combination (Figure 2).

Figure 2a shows the single effect of aeration and agitation including maximum, minimum and average predicted activities (confidence level, 95%). The predicted combined effects of the variables, given by a response surface plot, are shown in Figure 2b, while comparison between experimental and predicted data is reported in Table 2.

**Table 2:** Experimental results and predicted data by the model

Experiment (Thesis number)	Observed (U/L)	Predicted (U/L)
T1	92.29	98.89
T2	217.37	233.13
T3	221.17	233.13
T4	259.26	246.80
T5	218.53	230.21
T6	356.81	329.13
T7	381.19	360.67
T8	219.37	219.53
T9	247.48	283.13
T10	282.22	332.13
T11	92.01	66.85
T12	116.00	95.12
T13	109.95	95.13
T14	126.94	161.78
T15	92.17	98.89
T16	129.80	161.78
T17	142.98	161.78
T18	215.13	161.78
T19	375.69	360.47
T20	394.25	360.47

Lowest activity (ca. 92 U/L) was obtained at 0.5 vvm both at 200 (T1, T15) and 500 rpm (T11). Highest enzyme activity was achieved at 1.0 vvm and 300 rpm (T7, T19 and T20 average = 383.7 ± 7.8 U/L). In these conditions the predicted value was 360.47 U/L (Table 2). The optimal combination to obtain highest activity (373.0 U/L), as predicted by the model, was 1.1 vvm and 327 rpm (Figure 2b). This condition was not included in the experimental design and additional experiments, carried out accordingly, confirmed model reliability. Actually, the production of chitinolytic enzymes (378.3 ± 6.1 U/L) was not statistically different from the model prediction. No statistical difference was found also with the value measured at 1.0 vvm and 300 rpm (383.7 ± 7.8 U/L) as mentioned before (Table 2).

Accordingly to the model, enzyme activity increased up to 1.1 vvm and to 327 rpm; beyond these values, and regardless the combination chosen, it always decreased. In certain conditions decrease was dramatic (i.e. agitation > 400 rpm). It is worth noting that, exceeding these threshold values, the negative effect of agitation was much higher than that of aeration. This is particularly evident observing the slopes of the plots showed in Figure 2a.

These outcomes could be explained by several causes with possible synergisms. At low aeration and agitation, no sufficient oxygen was probably available for the fungal growth and consequent enzyme production. This was confirmed by the low K_L_a (Figure 1) and by the delay necessary to obtain the maximum activity (144 h instead of 72 h, data not shown).

Lack of oxygen availability did not explain the low activities recorded beyond the mentioned values in correspondence of which K_L_a was always high. In these cases, shear stress could be the principal cause of cell sufferance and consequent low enzyme production. In addition, the enzyme, already released in the cultural broth, could be partially inactivated by the shear forces. Negative effects of stirring were never demonstrated for chitinolytic enzymes but they are known for other secreted proteins and could be related to catalytic dysfunctions due to conformational changes carried out by mechanical forces [24-27].

The negative effect of the increased stirrer speed was so strong to be higher than that caused by low oxygen availability. In fact, lowest enzyme activity (ca. 92 U/L) was obtained both at 0.5 vvm and 200 rpm, leading to lowest K_L_a (18.3 h^-1^) and at 0.5 vvm and 500 rpm with rather high K_L_a (51 h^-1^). This effect was well evidenced in the response surface plot (Figure 2b).

Due to the scarce specific literature available, comparison of our results with those of other scientists is difficult. Some of our results were in contrast with those reported by Liu et al. [15] carried out by the OVT method. These authors correlated the production improvement with the increase of stirring with no mention to possible shear stress at high rates. Probably, in that case, the range of stirring tested (75 to 225 rpm in a 5 L bioreactor) was too narrow and highest value too low to consent observations of this phenomenon. By contrast, our work agrees with these authors to correlate reduction of enzyme activity with the increase of aeration [15].

Negative effects, on chitinolytic enzymes production, by mechanical stress due to increasing stirrer speed in CSTR, were also observed for *T. harzianum *[14,16].

### Bioprocess under optimized conditions

In view of process scale-up to the industrial level, the use of low aeration and agitation rates is preferable for energy saving. In this context, 1.0 vvm and 300 rpm were preferable than 1.1 vvm and 327 rpm with (ca. 10% less). Therefore, these have been considered as optimal conditions for the cultivation of *L. muscarium *in bioreactor and its production of chitinolytic enzymes. Detailed studies on bioprocess kinetics have been carried out accordingly.

**Figure 3:**
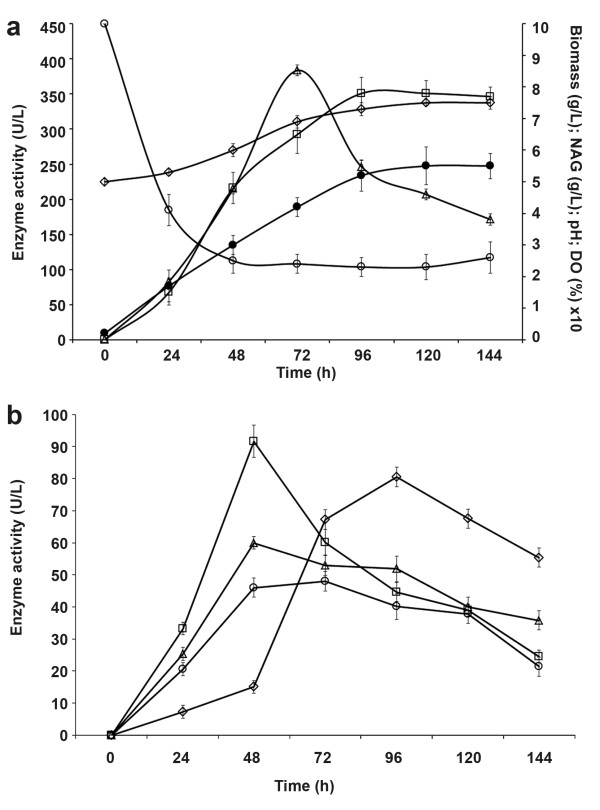
**Time course of bioprocess parameters by *L. muscarium *CCFEE 5003 cultivated in 2 L CSTR under optimized conditions**. (a) Total chitinolytic enzyme production (Δ), biomass (●), NAG (□), DO (○) and pH (◇). (b) Chitinolytic enzyme activities as revealed by S1 (◇), S2 (□), S3 (Δ) and S4 (○).

Figure 3a shows the time course of total chitinolytic activity, fungal biomass, dissolved oxygen (DO), pH and NAG production by *L. muscarium *CCFEE 5003 grown for 144 h under optimised conditions. Enzyme activity 383.7 ± 7.8 U/L peaked at 72 h to strongly decrease thereafter. Maximum biomass production was delayed (120 h) in relation to the enzyme activity. This was probably due to a late availability of nutrients produced by chitin hydrolysis as confirmed by the peak of NAG recorded at 96 h. pH increased from the initial value (5.5) up to ca. 7.5.

Detailed information, on the production of enzymes with different activity on chitin, was not obtained from the DNSA assay, revealing total chitinolytic activity, but using the 4-MU substrates (S1-S4). The time course of these different typologies of chitinolytic activities is shown in Figure 3b.

The pattern of enzymes produced was variable during the process in relation to the development of chitin hydrolysis. The enzymes with possible "endo-" or "exo-" activity (revealed by S2, S3 and S4) peaked at 48 h; while those hydrolysing the chitobiose (revealed by S1) peaked at 96 h. In the early stages of chitin hydrolysis, coarse polymers fragmentation was probably carried out by endo-type enzymes (i.e. the chitinases, E.C. 3.2.1.14) with the contribution of eso-type activities (i.e. the *N*-acetyl-hexosaminidases, E.C. 3.2.1.52). Later on, massive chitobiose and NAG production were obtained from oligomers and longer segments. The degradation of chitobiose requires presence of the chitobiase: this activity is grouped by the Enzyme Commission within the *N*-acetyl-hexosaminidases class.

**Figure 4:**
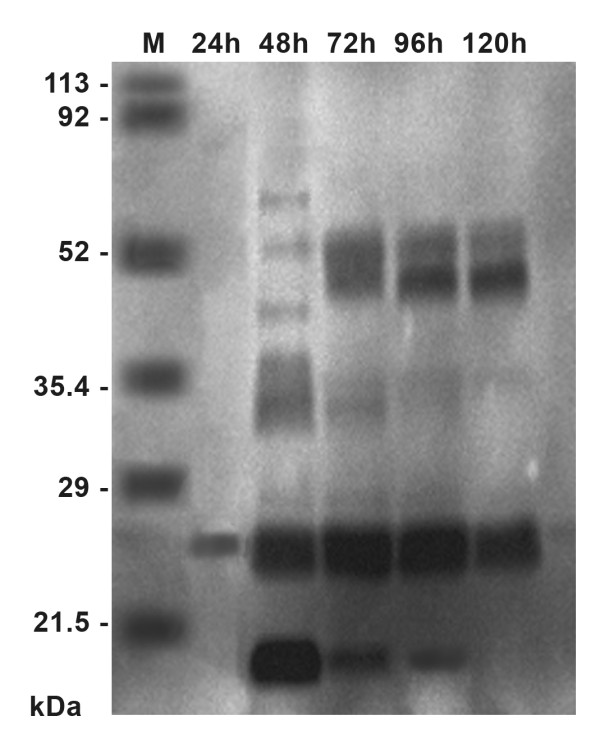
**Electrophoresis (PAGE) activity gel for chitinolytic enzymes produced by *L. muscarium * CCFEE 5003 cultivated in 2 L CSTR under optimized conditions**. Samples were samples taken every 24 h; molecular weight markers (21.5 - 113 kDa) are also shown.

The production of a complex and time-evolving pattern of chitinolytic enzymes by *L. muscarium *was confirmed when PAGE activity gels were obtained from fermentation broth samples taken at different times. These showed presence of various enzymes (at least 7) with different MWs (Figure 4). The first enzyme (MW of ca. 25 kDa) was revealed after 24 h, increased thereafter and was constantly present along the whole process. By contrast, presence of other enzymes (MW from ca. 20 to ca. 55 kDa) was not constant. The production of a diversified large array of chitinolytic enzymes, showing complete competence in chitin hydrolysis, is typical of parasitic fungi such as *Trichoderma *species. By contrast, saprophytes often present few activities with limited competence on chitin degradation [3,28,29].

It is worth noting that, due to the establishment of much more suitable conditions, *L. muscarium*, when cultivated in bioreactor after the optimization reported in this study, was able to release a number of chitinolytic enzymes higher than that obtained in shaken flasks as reported by Fenice and Gooday [3]. In addition, the total chitinolytic enzyme production increased by more than 25%.

## Conclusions

The Antarctic fungus *L. muscarium *CCFEE 5003 is a powerful chitinolytic organism never exploited at the industrial level. In this study we demonstrated its ability to growth and produce high levels of chitinolytic enzymes in CSTR. The optimization of the production process at laboratory level, carried out with a modern and efficient approach (RSM), permitted to obtain appreciable qualitative (increased number of enzymes) and quantitative (increased production) advantages. These are very important and promising assumptions in view of the development of an economically valuable industrial production.

## Material and methods

### Chemicals

Chitin (from crab shells), *N*-acetyl-D-glucosamine (NAG), *N*-*N*'diacetylchitobiose and *N*-*N*'-*N*"triacetylchitotriose, cellobiose, 4 methylumbelliferone (4MU); 4-methylumbelliferyl *N*-acetylglucosaminide (S1); 4-methylumbelliferyl β-D-*N, N'*-diacetylchitobioside (S2); 4 methylumbelliferyl *N, N', N"*-triacetylchitotrioside (S3) and 4-methylumbelliferyl *N, N', N", N"'*-tetraacetylchitotetraoside (S4) were from Sigma Chemicals (St. Louis, MO, USA); Malt Extract Agar (MEA) was from Oxoid (U.K.); Yeast Nitrogen Base (YNB) was from Difco (Detroit, MI, USA). All other chemicals were of analytical grade.

Colloidal chitin was prepared as already reported [30]. After swelling, chitin was re-suspended in water and autoclaved (121°C, 20 min). Glycol chitin was prepared as reported by Molano et al. [31].

### Microorganism and inoculum preparation

*Lecanicillium muscarium *Petch strain CCFEE 5003 (ex *Verticillium lecanii *Zimm strain A3), a fungus previously isolated from Antarctic moss [32], was stocked in the Culture Collection of Fungi from Extreme Environments (CCFEE:) of the Dipartimento di Scienze Ecologiche e Biologiche, University of Tuscia, Viterbo, Italy. During the study the strain was maintained and routinely sub-cultured on MEA slants. Inocula were produced as previously described [3].

### Bioreactor and fermentation conditions

The bioreactor used was 2-l (total volume) bench-top stirred tank reactor (STR) (Applikon Dependable Instruments, Schiedam, NL) filled with 1.2 l of medium. The fermentor was equipped with a top stirrer bearing two six-blade Rushton-type turbines (diameter 4.5 cm, blade width 1.4 cm, blade length 1.4 cm) and 2 baffles (width 1.4 cm). Air was injected through a perforated pipe sparger located under the bottom turbine. The following probes were installed on the top plate: dissolved oxygen sensor (Ingold, CH), double reference pH sensor (pHoenix Electrode Company, Houston, Texas, USA), PT 100 temperature sensor. Standard bioprocess conditions were as follows: inoculum size 0.5 × 10^6 ^conidia/ml; temperature 25°C; initial dissolved oxygen 100% of saturation non-controlled; initial pH 5.0 non-controlled; basal fermentation medium (BM), as already reported as optimal for the chitinolytic enzyme production by the fungus [8], was: YNB 1%, colloidal chitin 1%. Silicone antifoam 0,2% was added to BM. Stirrer speed and aeration were set as reported below. All media were sterilized in the bioreactor for 30 min at 121°C.

The fermentation parameters (temperature, pH and dissolved oxygen) were monitored by an adaptative/PID digital controller, ADI 1030 (Applikon Dependable Instruments, Schiedam, The Netherlands).

Samples were taken every 24 h and, after centrifugation (10,000 g for 10 min), supernatants used as enzyme solutions for the enzyme assay determination.

Influence of the agitation on the mycelial growth and enzyme productions was evaluated at 200, 300, 400 and 500 rpm (corresponding to tip speed of 47.12, 70.68, 94.28 and 117.81 cm/s, respectively); the effect of aeration was tested at 0.5, 1.0 and 1.5 vvm. Both these parameters were optimised by RSM.

### Measure of K_L_a

The volumetric mass-transfer coefficient (K_L_a) of the bioreactors was determined by the static method of gassing out of Wise [33] using BM at different combinations of aeration rates (0.5, 1.0 and 1.5 vvm) and stirrer speeds (200, 300, 400 and 500 rpm) as suggested by the model. Tests were carried out at 25°C.

### Analytical methods

For the RSM the overall chitinolytic activity was determined by the method of dinitrosalicylic acid (DNSA), using *N*-Acetyl-D-glucosamine for standard curve, as previously reported [8]. For the production of chitinolytic enzymes under optimised condition activities were detected also by the release of 4 methylumbelliferone from 4MU substrates, using 4 methylumbelliferone for the standard curve, as reported by McCreath and Gooday [34].

Under the assay conditions, one unit (U) of enzyme activity was defined as the amount of enzyme which released 1 μmol per ml per min.

The mycelial growth was measured as reported by Fenice et al. [8].

### Electrophoresis and activity gels

Presence of various chitinolytic enzymes and their molecular weight (MW) was also determined using one-dimensional activity polyacrylamide gel electrophoresis (PAGE) following the procedure of Trudel and Asselin [35].

Markers (range 15-150 kDa) were from Bio-rad Laboratories (Ca, USA).

### Experimental factorial design by RSM

Effect of the combined action of aeration and agitation on the production of chitinolytic activity and K_L_a (response variables) was optimised by a D-optimal design, with the following independent variables (factors)

$${\text{X}}_{\text{1}}=\text{ Aeration }\left(\text{vvm of air}\right)$$

$$X_{\text{2}}=\text{ Agitation }\left(\text{rpm}\right)$$

The above dimensional independent variables were coded as dimensionless terms by the following equation:

$${\text{X}}_{i}=\;\left({\text{A}}_{i}-{\text{A}}_{0}\right)/\text{Δ}\text{A}\;\;\;\text{i}=\text{ 1},\text{ 2}$$

where X*_i _*is a coded value and A*_i _*is the actual value of the variable, A*_0 _*is the actual value of the same variable at the centre point and ΔA is the variable step change.

The range of the variables is given in Table 3.

**Table 3:** Experimental setup combining aeration and agitation as suggested by the model

Experiment (Thesis number)	Aeration (vvm)	Stirrer speed (rpm)
T1; T15	0.5	200
T2; T3	1.5	200
T4	1.0	200
T5	0.5	300
T6	1.5	300
T7; T19; T20	1.0	300
T8	0.5	400
T9	1.5	400
T10	1.0	400
T11	0.5	500
T12; T13	1.5	500
T14; T16; T17; T18	1.0	500

Data were subjected to analysis of variance (ANOVA) and fitted according to a second-order polynomial model shown by:

$$Y=\beta o+\;\sum^{\text{​}}\beta_{i}X_{i}+\;\sum^{\text{​}}\beta_{ii}X_{i}^{2}+\;\sum^{\text{​}}\beta_{ij}X_{i}X_{j}$$

where *Y *is the predicted response variable, βo is the intercept, βi and βii linear coefficient and quadratic coefficient, respectively, βij is the interaction coefficient and *X*i and *X*j are the coded forms of the input variables. To estimate the impact of single independent variables on the response, regardless of the presence of the other factors, main effects were calculated using:

$$Y=\text{β}\text{o}+{\text{β}}_{\text{i}}X_{\text{i}}+{\text{β}}_{\text{ii}}{\text{X}}_{\text{i}}^{2}$$

Statistical examination of results and response surface study were carried out by the MODDE 5.0 software (Umetrics AB, Sweden).

Fermentations under optimal conditions for best enzyme production, as suggested by the model, were carried out in triplicate in subsequent experiments.

## Competing interests

The authors declare that they have no competing interests.

## Authors' contributions

MF and PB carried out the design of this study. MF overviewed fermentations, performed determinations and data analysis. LS was responsible of fermentations and performed determinations. PB performed data analysis. MF, PB, LS and FF participated in writing and critical manuscript review. All authors have read and approved the manuscript.
